# Molecular basis for the reproductive division of labour in a lower termite

**DOI:** 10.1186/1471-2164-8-198

**Published:** 2007-06-28

**Authors:** Tobias Weil, Michael Rehli, Judith Korb

**Affiliations:** 1 Biologie I, Universität Regensburg, Universitätsstrasse 31, 93040 Regensburg, Germany; 2 Hämatologie und Onkologie, Klinikum der Universität Regensburg, Franz-Josef-Strauss-Allee-11, 93053 Regensburg, Germany

## Abstract

**Background:**

Polyphenism, the expression of different phenotypes with the same genetic background, is well known for social insects. The substantial physiological and morphological differences among the castes generally are the result of differential gene expression. In lower termites, workers are developmentally flexible to become neotenic replacement reproductives via a single moult after the death of the founding reproductives. Thus, both castes (neotenics and workers) are expected to differ mainly in the expression of genes linked to reproductive division of labour, which constitutes the fundamental basis of insect societies.

**Results:**

Representational difference analysis of cDNAs was used to study differential gene expression between neotenics and workers in the drywood termite *Cryptotermes secundus *(Kalotermitidae). We identified and, at least partially cloned five novel genes that were highly expressed in female neotenics. Quantitative real-time PCR analysis of all five genes in different castes (neotenics, founding reproductives, winged sexuals and workers of both sexes) confirmed the differential expression patterns. In addition, the relative expression of these genes was determined in three body parts of female neotenics (head, thorax, and abdomen) using quantitative real-time PCR.

**Conclusion:**

The identified genes could be involved in the control and regulation of reproductive division of labour. Interestingly, this study revealed an expression pattern partly similar to social Hymenoptera indicating both common and species-specific regulatory mechanisms in hemimetabolous and holometabolous social insects.

## Background

Social insects (termites and social Hymenoptera, such as ants, some bees, and wasps) are the exemplars of social life. They are characterized by a reproductive division of labour in which only a few individuals within a colony reproduce (queen/s, and king/s in termites), while the large majority helps in raising offspring (workers, in termites additionally soldiers). This caste system is a result of phenotypic plasticity; i.e. different castes generally arise from environmentally induced differential gene expression [1-3].

**Figure 1:**
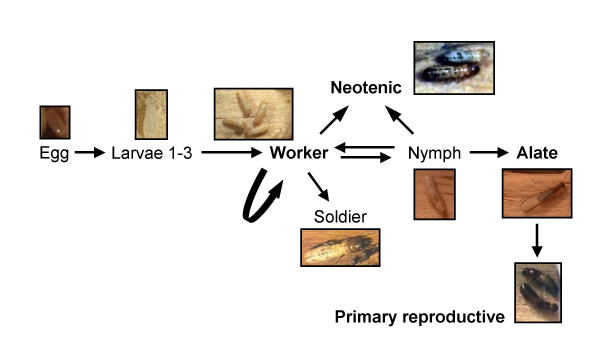
**Developmental Pathways of *Cryptotermes secundus***. Caste differentiation in lower termites reflects larval polphenism as reproductives are the only adults. Workers which develop from totipotent eggs have the potential (i) to remain workers by moulting stationarily (moulting without change of the external morphology) or regressively (returning to morphological characters of an earlier instar), (ii) to develop into sterile soldiers that defend the colony, (iii) to become alates that disperse from the nest and found an own colony as primary reproductives, or (iv) to reproduce in the natal as neotenic replacement reproductives when the same-sex reproductive of the colony dies. The development from a worker into a neotenic requires only a single moult. Bold letters indicate castes used for analysis.

In termites, caste polymorphism is the result of a highly flexible postembryonic development which is especially pronounced in wood-nesting species. Here, workers (sometimes also called pseudergates, false workers or helpers due to their flexible development; [4] develop from totipotent eggs and have the possibility (i) to become winged sexuals (alates) that disperse from the nest and found their own colony as primary reproductives; (ii) to reproduce in the natal nest as neotenic replacement reproductives when the same-sex reproductive of the colony dies, or (iii) to develop into sterile soldiers that defend the colony (Figure.1). The development into each of these castes requires different numbers of moults; several for alates, one for neotenics and two for soldiers. As an alternative, individuals can remain as workers in the nest by moulting stationarily (moulting without change of the external morphology) or regressively (returning to morphological characters of an earlier instar). Research in termites so far concentrated on the development of soldiers [5-9]. Termite soldiers are a unique caste with no equivalent in other social insects [10,11]. A comparison of differential gene expression between reproductives and workers may, however, allow the identification of common principles and differences in the regulation of reproductive division of labour between social insect taxa.

We specifically addressed the question of what characterizes a queen by comparing gene expression profiles between workers and female reproductives in the drywood termite *Cryptotermes secundus*. In termites, neotenic replacement reproductives are especially suited for this purpose because they differ from workers only by traits linked to reproduction, while confounding traits that are developed by winged sexuals for the dispersal process (e.g. compound eyes, wings) are not expressed. Our analysis revealed a number of interesting genes that are primarily expressed in neotenic replacement reproductives and may be involved in processes controlling or maintaining the reproductive division of labour.

## Results

### Identification of caste-specific transcripts in female neotenics

**Figure 2:**
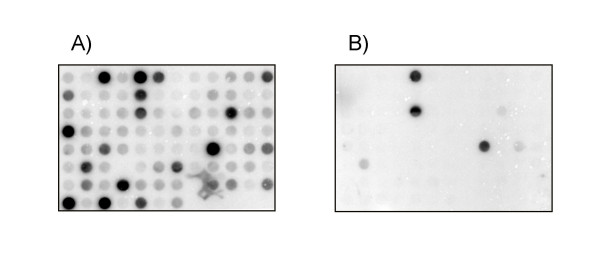
**Reverse Dot Blot of cloned RDA fragments**. A representative reverse dot blot of the RDA difference products is shown. Cloned inserts of randomly picked clones were PCR-amplified, denatured and dot-blotted onto two duplicate nylon transfer membranes and hybridized to radioactively labelled female neotenic (A) and worker (B) representations.

**Figure 3:**
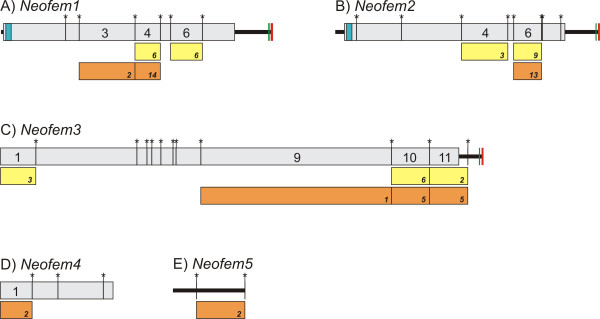
**Physical map of *Neofem *genes**. Physical maps depicting *Neofem* 1–5 genes (A-E) and the isolated RDA fragments; bold line: cDNA strand; grey bar: open reading frame; numbers; *Dpn*II fragment; yellow bar: *Dpn *II fragment obtained from the first RDA; orange bar: *Dpn *II fragment obtained from the second RDA; turquoise bar: signal peptide; green bar: poly-adenylation-signal; red bar: poly-A-tail; asterisk: *Dpn *II restriction site; italic numbers: number of individual sequenced fragments.

The limited publicly available information on genome or cDNA sequences of the drywood termite *Cryptotermes secundus *(Kalotermitidae) restricts the number of possible screening techniques for differential gene expression analysis. We chose to compare termite castes using the representational difference analyses of cDNA (cDNA-RDA) approach because it is independent of sequence knowledge and requires relatively small amounts of mRNA. To identify genes that are specifically expressed in female neotenics, we initially performed a cDNA-RDA using female neotenics as tester cDNA and workers of both sexes as driver cDNA. The difference product of the third round was shotgun cloned and 187 randomly picked clones were validated using reverse dot blot hybridization with labelled tester and driver cDNAs. A representative dot blot hybridisation of representational difference products is shown in Figure 2. Thirty five out of 38 sequenced fragments with highly specific signals in reverse dot blot hybridization were derived from termites and most likely belonged to three independent genes that were named *Neofem1 – Neofem3*. To identify additional fragments we performed a second RDA where we suppressed the seven initially identified, highly overrepresented fragments by adding them in excess to the driver population. Additional 192 randomly selected clones were picked and analysed as above. Sequencing of 52 clones revealed 8 novel fragments including five fragments that most likely belonged to three different genes (*Neofem4, Neofem5 *and a putative transferrin homolog). Seven sequences were most likely of non-termite origin. Mapping of individual fragments was done by a series of inter-fragment PCRs and by 3'- and 5'-RACE-PCRs, confirming the initial assignment of the identified fragments to six genes. Complete transcripts were obtained for two genes (*Neofem1 and Neofem2*), partial 3'- or 5'-sequences were obtained for all other genes. Overrepresentation of the putative transferrin cDNA ([GeneBank: EF029058]) could not be validated by quantitative real-time PCR (qRT-PCR, data not shown). Physical maps of the remaining five genes indicating the location and number of cloned RDA fragments are shown in Figure 3. The *Neofem1 *gene encodes a putative polypeptide of 558 amino acids. Its N-terminus comprises a signal peptide suggesting that the *Neofem1 *gene product is secreted. A comparative sequence analysis using the BLAST-X algorithm suggests similarity to genes of the esterase-lipase family, in particular to genes of *Tribolium castaneum *and *Apis mellifera *that are similar to an uncharacterised *Drosophila *gene ortholog. The putative 532 amino acid gene product of the *Neofem2 *gene also contained a signal peptide. Similarity searches identified homologies of the *Neofem2 *gene product to members of the glycosyl hydrolase family1, in particular for a beta-glucosidase gene of the termite *Neotermes koshuensis *[12] and a male-specific β-glycosidase of the Madeira cockroach *Leucophaea maderae *[13]. The partial sequence of *Neofem3 *showed the highest sequence similarity to the Vitellogenin 1 precursor (Vg-1) sequence of the American cockroach *Periplaneta americana *[14] which serves as a precursor of egg-yolk proteins. The putative *Neofem4 *gene product is closely related to family 4 cytochrome P450 enzymes (CYP4) from arthropods, with highest similarities to CYP4U1 from the Australian termite *Coptotermes acinaciformis *[GenBank:AAC03111] and to CYP4C1 of *Blaberus discoidalis *[15]. No homologies were found for the *Neofem5 *gene fragment. Table 1 summarizes the sequence analysis of all these genes. Complete nucleotide sequences were submitted to GenBank [GenBank:EF029054 – EF029059].

**Table 1:** 

Gene	Size (bp)	No. of clones	Identity match by BLASTX [species]	Accession no.	Local identity (%)	Score (bits)	*e*-value
*Neofem1*	1970	28	PREDICTED: similar to CG4382-PA [*Tribolium castaneum*]	XP_974072	47	520	1E-145
			PREDICTED: similar to CG4382-PA [*Apis mellifera*]	XP_393293	46	493	1E-137
			juvenile hormone esterase [*Aedes aegypti*]	EAT39446	47	485	3E-135
*Neofem2*	1918	25	beta-glucosidase [*Neotermes koshunensis*]	BAB91145	50	510	8E-143
			male-specific beta-glycosidase [*Leucophaea maderae*]	AAL40863	48	504	7E-141
			PREDICTED: similar to CG9701-PA [*Tribolium castaneum*]	XP_972437	48	475	3E-132
*Neofem3*	3502	22	Vitellogenin 1 precursor (Vg-1) [*Periplaneta americana*]	Q9U8M0	32	592	3E-167
			Vitellogenin [*Athalia rosae*]	BAA22791	29	512	4E-143
			Vitellogenin 2 precursor (Vg-2) [*Periplaneta americana*]	Q9BPS0	28	449	5E-124
*Neofem4*	817	2	family 4 Cytochrome P450 [*Coptotermes acinaciformis*]	AAC03111	46	270	7E-71
			Cytochrome P450 4C1 (CYPIVC1) [*Blaberus discoidalis*]	P29981	38	217	4E-55
			Cytochrome P450 [*Aedes aegypti*]	EAT35570	40	550	9E-55
*Neofem5*	525	2	PREDICTED: similar to guanylate cyclase OlGC-R2 [*Danio rerio*]	XP_688499	52	32,2	8,70

**Description**: BLASTX results that show best hits against the non-redundant NCBI database, including species in [], GenBank accession number, local identity (%), score (bits) and E-score value. Further the sequence length (*Neofem1 *and *Neofem2 *= full length cDNAs; *Neofem3 *– *Neofem5 *= partial cDNAs) and the number of cDNA clones from RDA are shown.

### Quantitative expression analysis of the *Neofem*1–5 genes

**Figure 4:**
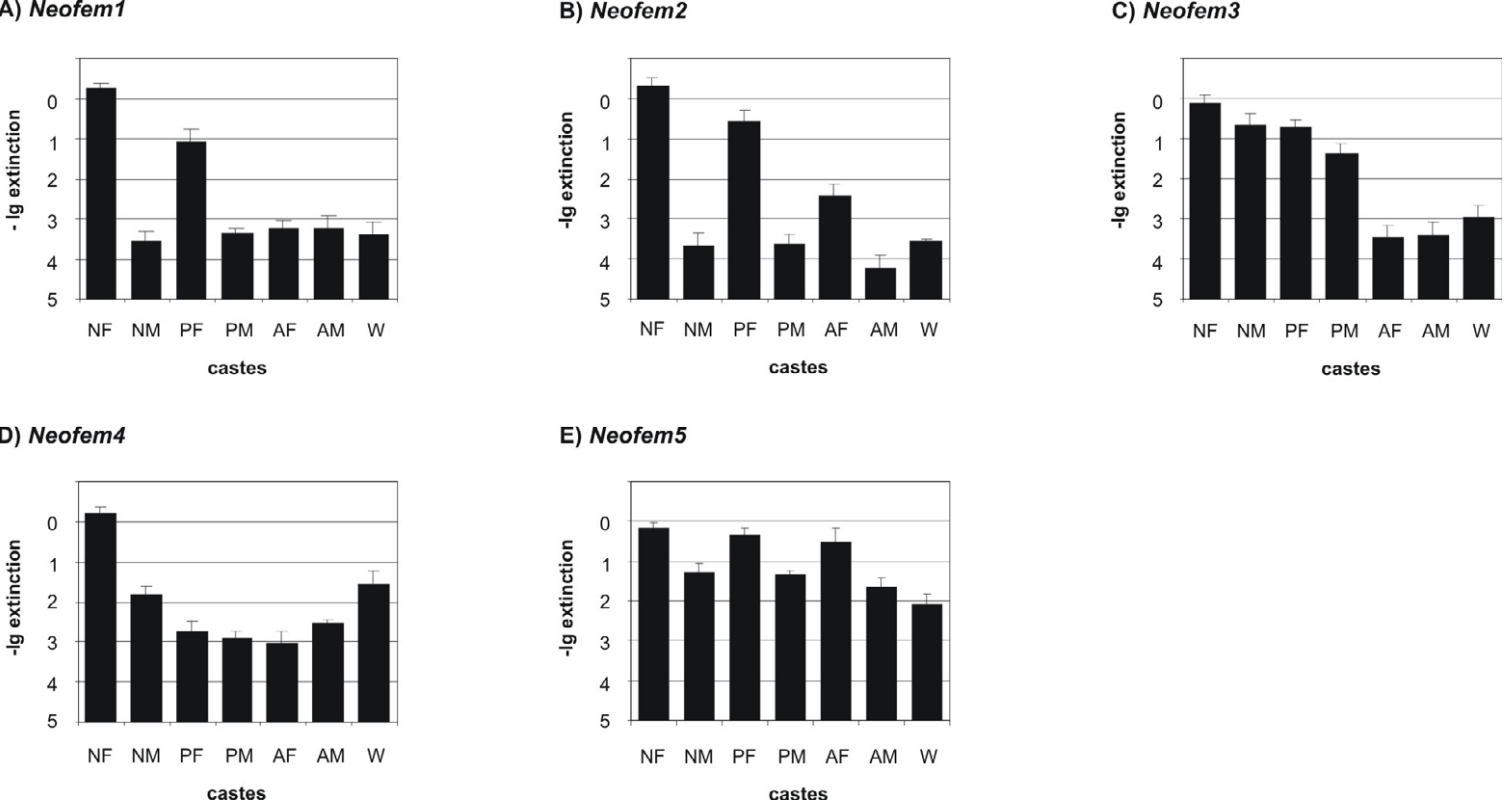
**Quantification of *Neofem *genes in different castes**. Relative expression levels of *Neofem *1–5 genes (A – E) in different castes measured by qRT-PCR. The Y-axis is on negative log_10 _scale indicating the gene expression levels and the calculated errors (SD), for female neotenics (NF), male neotenics (NM), female primary reproductives (PF), male primary reproductives (PM), female alates (AF), male alates (AM) and workers (W) of both sexes.

To validate and further analyse the expression of *Neofem*1–5 genes, we performed qRT-PCR using RNA-samples derived from different termite castes (neotenics, primary reproductives, winged sexuals and workers of both sexes). To be able to normalize the expression data, we initially cloned gene fragments of putative house keeping genes (*18S rRNA*, *β-actin *and *hexamerin*) and designed primers for qRT-PCR. The suitability of putative reference genes was evaluated by using the *BestKeeper *software [16]. The comparison revealed that *18S rRNA *was the most stable reference gene (N = 21, r^2 ^= 0.60, P = 0.001). Figure 4 shows relative expression levels of the five *Neofem *genes that met the selection criteria of the initial RDA – their expression was generally much higher in female neotenics as compared with workers. In line with the order of appearance and the fragment abundance in the initial RDAs, expression levels of the *Neofem1 – Neofem3 *genes in female neotenics were up to four orders of magnitude higher in female neotenics than in workers. As expected, the difference in gene expression was less pronounced in the two genes identified exclusively in the second RDA (*Neofem4 – Neofem5*). While *Neofem1 *(homologous to esterase-lipase) and *Neofem2 *(homologous to glycosidase) genes were almost exclusively expressed in female reproductives (neotenics and primaries), the gene homologous to vitellogenin (*Neofem3*) was expressed in all reproductives. Expression of the P450 homolog (*Neofem4*) was highest in female neotenics. The unknown transcript (Neofem5) was detected in all castes and showed highest expression in females.

**Figure 5:**
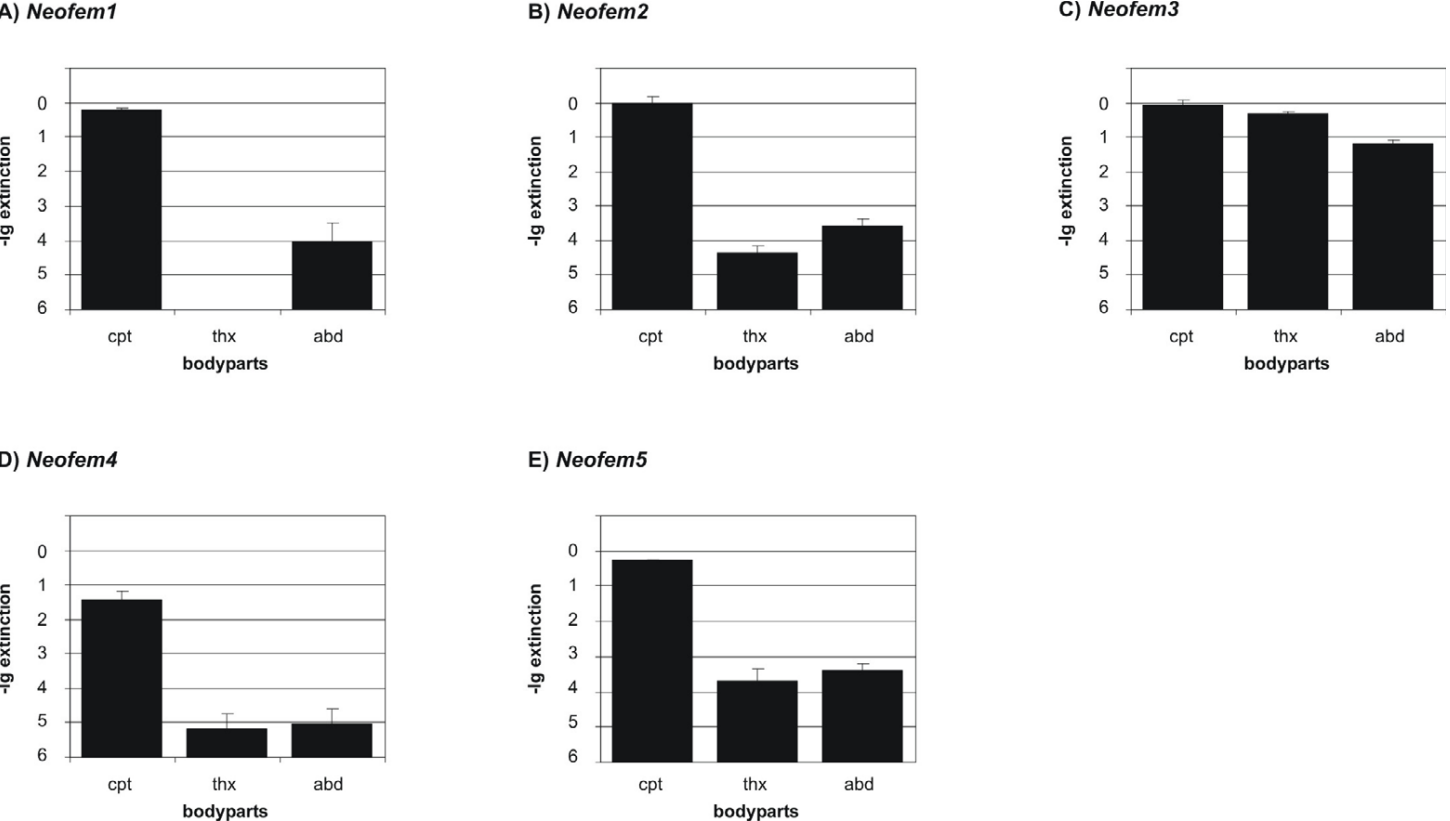
**Quantification of *Neofem *genes in different body parts**. Relative expression levels of *Neofem *1–5 genes (A -E) for different body parts measured by qRT-PCR. The Y-axis is on negative log_10 _scale indicating the gene expression levels and the calculated errors (SD). cpt: caput, thx: thorax and abd: abdomen.

To determine, where the five neotenic-specific genes are expressed in female neotenics, total RNA was prepared from different body parts (head/caput, thorax, and abdomen) and was analysed by qRT-PCR. As shown in Figure 5, four of the five genes were expressed primarily in the termite head. The gene *Neofem3 *was detected almost equally in all three body parts.

## Discussion

The ability of *Cryptotermes secundus *workers to develop into neotenic replacement reproductives after a single moult offers the unique possibility to study differential gene expression during caste differentiation. In this study, we compared the transcriptomes from female neotenics and workers of both gender using RDA to identify novel neotenics-specific transcripts.

We were able to identify five genes that were highly overrepresented in female neotenics of the drywood termite *C. secundus*. Four of these five genes were overexpressed in the head. Expression of the genes *Neofem1 *and *Neofem2 *of *C. secundus *was highly specific for female reproductives. Both genes are predicted to encode secretory proteins that are specifically expressed in the heads of female neotenics. The open reading frame of *Neofem1 *encodes a putative esterase-lipase which shows the highest similarity to yet uncharacterized proteins of the red flour beetle *Tribolium castaneum *and the honey bee *Apis mellifera *which are putative orthologs of the *Drosophila *protein CG4382-PA. In addition, two juvenile hormone esterases (JHE) of the mosquito *Aedes aegypti *are closely related to the *Neofem1 *protein sequence. Most interestingly, the *Apis mellifera *homolog (GB16889, [GenBank: XP_393293]) was found in the brain of adult female worker honey bees [17] and is closely related to a moth integumental carboxyl/cholinesterase which is implicated in pheromone processing [17,18].

*Neofem2 *showed highest similarity to a digestive β-glycosidase from the salivary glands of the termite *Neotermes koshuensis *[12]. Insect glycosidases are known to include, amongst others, digestive and pheromone degrading enzymes [12,13]. However, the lack of expression in males suggests a sex specific function. Thus *Neofem2 *is presumably not a digestive enzyme. Rather the close match to Lma-p72 protein of the Madeiran cockroach [13], which is sex specifically expressed in the abdominal glands of male cockroaches to attract females, may indicate a pheromonal function.

The *C. secundus Neofem4 *protein is closely related to family 4 cytochrome P450 enzymes (CYP4) from arthropods, with highest similarities to an uncharacterized termite CYP4 from *Coptotermes acinaciformes *and to CYPIVC1 from *Blaberus discoidalis *(Blattodea; [19]). Cytochrome P450 enzymes of insects are generally associated with the metabolism of endogenous substrates or hormones, and with detoxification (summarized by Feyereisen [20]). In termites and social Hymenoptera, some cytochrome P450 enzymes are expressed in a caste specific manner [3,6,21-23]. Contrary to these studies on Hymenoptera and on *Coptotermes acinaciformes *that all revealed highest expression levels in non-reproducing castes, *Neofem4 *of *C. secundus *was overexpressed specifically in female neotenics. In termites, cytochrome P450 enzymes are involved in metabolic pathways (*C. acinaciformis*) or insecticide resistance (*Mastotermes darwinensis*) [6,24]. However, the specific expression of *Neofem4 *in the head of female neotenics suggests that *Neofem4 *is involved in the metabolism of endogenous substrates like ecdysteroids or JH rather than insecticide resistance.

The gene *Neofem3 *is the only gene that is distributed almost equally in all body parts of female neotenics. It showed highest similarities to insect vitellogenins (Vgs), specifically to Vg1 of the American cockroach *Periplaneta americana *and a Vg of the turnip sawfly *Athalia rosae*. In most insect species vitellogenins are synthesized extraovarially in female fat body cells as large precursor proteins of vitellin (the major yolk protein of insects). Vgs are secreted into the haemolymph and then incorporated into developing oocytes [25,26]. High expression levels of Vg in female reproductives (primaries and neotenics) were expected because of their ovarian activity. The elevated Vg expression in male reproductives may be explained by the function of Vgs as storage proteins [25]. Recently it was shown that functionally sterile nursing honey bee workers utilize vitellogenin to produce royal jelly to feed larvae [26]. The above findings suggest that an ancestral reproductive protein, Vg, was repeatedly co-opted in different social species to serve different functions in different castes. Thus, Vg seems to function as an important developmental protein.

## Conclusion

We isolated and characterized five genes that were up-regulated in female replacement reproductives compared to non-reproducing workers of the drywood termite *Cryptotermes secundus *(Kalotermitidae). Interestingly, potential homologues of some of these genes appear to be expressed in different insect species, hemimetabolous as well as holometabolous, in a caste- and species-specific manner. Especially, pheromone-processing genes and Vg emerge as major players that were repeatedly exploited in social evolution of insect societies.

## Methods

### Chemicals

All chemical reagents used were purchased from Sigma-Aldrich (Taufkirchen, Germany) unless otherwise noted. Oligonucleotides were synthesized either by Metabion international AG (Martinsried, Germany) or by Carl Roth GmbH (Karlsruhe, Germany). Sequences of all Oligonucleotides are given in Additional file 1.

### Termites

Complete termite colonies (*Cryptotermes secundus*) were collected in mangroves around Darwin (NT, Australia) and held in climate chambers at 27°C and a relative humidity of 70% (for details see Korb and Schmidinger [27]). Primary reproductive and alates were taken from these colonies.

To obtain neotenic reproductives, big colonies were split and groups of at least 15 workers were placed together in new *Pinus radiata *wood blocks (16 × 4 × 4 cm^3^). After about two weeks neotenic reproductives developed which were removed together with two workers. The sex of the neotenics was determined by their sex-specific morphology as described by Grassé [28].

### RNA preparation

Total RNA from different castes and developmental stages was prepared using the RNAwiz™ solution (Ambion). Poly(A)mRNA was enriched using the MicroPoly(A)Purist™ Kit (Ambion) according to the manufacturer's recommendations. RNA purity and integrity were checked by agarose gel electrophoresis and by UV/Vis spectrometry.

### Representational difference analysis

Double-stranded cDNA was prepared by reverse transcription of 2 μg poly(A) mRNA using the Universal Riboclone^® ^cDNA Synthesis System (Promega). RDA was performed essentially as described by Heinz et al. [29]. Briefly, the driver representation consisted of cDNA generated from the pooled mRNA of 25 *Cryptotermes secundus *workers. This representation was subtracted from tester cDNA representation of the mRNA repertoire of 11 *C. secundus *female replacement reproductives. After three rounds of subtraction (driver excess: 50 ×, 400 × and 10.000 × in successive rounds) and amplification, the entire third difference product was gel-extracted and "shotgun"-cloned into the *BamH *I restriction site of the pZErO-2 vector (Invitrogen) according to the manufacturer's instructions. To check for specificity of the difference product, inserts of randomly picked clones were PCR-amplified from single bacterial colonies utilizing vector-specific primers. The PCR products were denatured with 3 M NaOH for 30 min at room temperature and blotted in duplicates on two separate nylon membranes (Magna NT, 0.22 μm; MSI) in 20 × SSC using a vacuum dot blot manifold (Schleicher und Schuell). After UV-cross-linking, one blot was hybridized to driver (worker), the other blot to tester (female neotenics) cDNA representation, which had been labelled radioactively with Klenow fragment (Roche Biochemicals) according to standard protocols. After stringent washing, membranes were exposed to a Molecular Dynamics Storage Phosphor Screen overnight and scanned on a Typhoon 9200 Variable Mode Imager (Amersham Pharmacia). An additional RDA was performed starting with the first difference product of the first RDA. The procedure was modified by adding *Dpn *II fragments of three genes obtained from the first round to achieve additional *Dpn *II fragments. Here two additional rounds of subtraction (driver excess: 400 × and 5.000 × in successive rounds) and amplification were performed. Products were cloned and analysed as above.

### RNA ligase-mediated 5'- and 3'-Rapid Amplification of c DNA Ends (RACE)-PCR

To obtain complete cDNAs of genes corresponding to the identified RDA fragments, 5'- and 3'-end RACE-PCRs and inter-fragment PCRs were performed. One μg of total RNA from female neotenics was used for cDNA synthesis with the FirstChoice™ RLM-RACE Kit (Ambion). The outer and inner primers for nested PCRs of the genes *Neofem *1–5 and the putative transferrin were derived from gene-specific PCR fragments obtained during the RDA (sequences are given in Additonal file 1). They were used to amplify 5'- and/or 3'-cDNA fragments. PCR products were cloned into pCR2.1-TOPO vector (TOPO Cloning Kit, Invitrogen) and inserts from several individual plasmid-containing bacterial colonies were sequenced (by GENEART, Regensburg, Germany). Oligonucleotide primers for full-length cDNA amplification were designed according to sequence alignments. PCR products were cloned into pCR2.1-TOPO (TOPO Cloning Kit, Invitrogen) and subsequently sequenced.

### Quantitative real-time PCR

Total RNA (1 μg) was reverse transcribed using Superscript II RT (Invitrogen) and Random Decamers (Ambion). qRT-PCR was performed on a Mastercycler^® ^ep *realplex *(Eppendorf) using the QuantiTect SYBR green PCR Kit (Qiagen) according to the manufacturer's instructions. Primers are given in Additional file 1. Melting curves were analyzed to control for specificity of the PCR reactions. Expression data for genes were normalized for expression of the *18S rRNA*. The relative units were calculated from a standard curve plotting 3 different concentrations of log dilutions against the PCR cycle number (CP) at which the measured fluorescence intensity reached a fixed value. Values represent mean +SD of three independent experiments.

### Sequence analysis

Alignments were performed using the software Gene Runner Version 3.05 (Hastings Software Inc.) and BioEdit Version 7.0.1 (Tom Hall Isis Pharmaceuticals, Inc.). BLAST-X database [30] searches were conducted to establish cDNA clone identity.

## Authors' contributions

TW performed the study. TW, MR and JK designed the study and drafted the manuscript. MR and JK coordinated the study and acquired funding. All authors read and approved the final manuscript.

## Supplementary Material

Additional file 1 List of used Oligonucleotides. Oligonucleotides used in this study for detection of *Neofem *expression in qRT-PCR, RACE PCR, RDA and standard applications. Click here for file
