# Supplementary material for: Molecular basis for the reproductive division of labour in a lower termite
Source: BMC Genomics. 2007 Jun 28;8:198. doi: 10.1186/1471-2164-8-198 (PMC1988835; doi:10.1186/1471-2164-8-198)
Supplement: Additional file 1 — List of used Oligonucleotides. Oligonucleotides used in this study for detection of Neofem expression in qRT-PCR, RACE PCR, RDA and standard applications. [file 1471-2164-8-198-S1.doc]

| **Oligonucleotides** | **Sequence (5’ – 3’)** |
| --- | --- |
| ***Real time-Oligonucleotides*** |  |
| LC-Neofem1-S | CTA TTC TTC ATA CCC ACA CTA GCC CC |
| LC-Neofem1-AS | CTT GGG AGT TCG CTG TTG CC |
| LC-Neofem2-S | GAT TAC CAC AAT TTT CCA CGG AGG |
| LC-Neofem2-AS | CAC GAG AGT CAC TCC TGT GTC CC |
| LC-Neofem3-S | CTC TGA ATC TGA CGA AAC AGC G |
| LC-Neofem3-AS | GCT TCT TTC CAT ATG CAG AGG G |
| LC-Neofem4-S | CAA TCA GCG CTT CAT CCC AC |
| LC-Neofem4-AS | TAT CTC GCT TGT TGC CTC CC |
| LC-Neofem5-S | TCT CCG ACA TCT ATC TGT GCC G |
| LC-Neofem5-AS | TGC AAC TAC CAC GAC GAC GG |
| LC-trans-S | GAC TTC GAG CTC CTG TGT CCT G |
| LC-trans-AS | ACA GTC TGA AGA GGT CCG GTC G |
| LC-18-S | AGG TGA AAT TCT TGG ATCGTC GC |
| LC-18-AS | AGT CAT CGG AGG AAC TTC GGC |
|  |  |
| ***RACE-Oigonucleotides*** |  |
| ***5’ RACE*** |  |
| outer-Neo1-5’ | TGT AGT AAT AGA GAG GTG CAC CGC |
| inner-Neo1-5’ | TCA TAG AAG AAT GCT ATT GGA GCC |
| outer2-Neo1-5’ | AGA AAC GAA GAA CCA CTC ATG GC |
| inner2-Neo1-5’ | TAA TAG AGC CAA CAC TGT AGC CTG C |
| outer-Neo2-5’ | TTT GTT GTG TAT TGA TTG AGA CCG |
| inner-Neo2-5’ | GTA ATC GCG ATT TTA GGT AGC |
| outer-Neo3-5’ | ATT TCT GTT CGA AGT AGT TAA GCG C |
| inner-Neo3-5’ | TAC GAG TTA CAG ATG CAA ACA GGG |
| outer-Neo4-5’ | TAT CTC GCT TGT TGC CTC CC |
| inner-Neo4-5’ | TAC ATG CAG TGG GAT GAA GCG |
| outer-Neo5-5’ | TGC AAC TAC CAC GAC GAC GG |
| inner-Neo5-5' | ACA GAT AGA TGT CGG AGA GGC G |
|  |  |
| ***3’ RACE*** |  |
| outer-Neo1-3' | TTA TAT CTC ATC TGA GCG GTG CAC |
| inner-Neo1-3' | ACT ACA AGT TCT CAT ACC AAG GCC G |
| outer- Neo2-3' | GGA GTC TGC TGG ACA ACA TGG |
| inner-Neo2-3' | GGG AAA TTG TCA TCA CTG AAA ATG |
| outer-Neo3-3' | TAC ATT GGA AGC AGC GAC AGC |
| inner-Neo3-3' | TGG TCC AGT GTG AAA GAA GCG |
| outer-Neo4-3' | CAA TCA GCG CTT CAT CCC AC |
| inner-Neo4-3' | CAA CAA GCG AGA TAC CTG GCC |
| outer-Neo5-3' | TCT CCG ACA TCT ATC TGT GCC G |
| inner-Neo5-3’ | TGG ACG ACG TGA TGG TAG GG |
| outer-trans-3' | ACA GTC TGA AGA GGT CCG GTC G |
| inner-trans-3' | AAC TCT ACA GCA AGC GAC CGG |
|  |  |
| ***Standard - Oligonucleotides*** |  |
| R-Bgl-24 [1] | AGC ACT CTC CAG CCT CTC ACC GCA |
| R-Bgl-12 [1] | GAT CTG CGG TGA |
| J-Bgl-24 [1] | ACC GAC GTC GAC TAT CCA TGA ACA |
| J-Bgl-12 [1] | GAT CTG TTC ATG |
| N-Bgl-24 [1] | AGG CAA CTG TGC TAT CCG AGG GAA |
| N-Bgl-12 [1] | GAT CTT CCC TCG |
| Neo1-S1 | TAA TGT TGC ACA TGG TGT CAC C |
| Neo1-S2 | AAG CAC TGT CTG TCA TTC TAA ACT CCT |
| Neo1-S3 | TTA TAT CTC ATC TGA GCG GTG CAC |
| Neo1-S4 | ACT ACA AGT TCT CAT ACC AAG GCC G |
| Neo1-AS1 | GAC AGA CAG TGC TTT ATG TGA AAC TCC |
| Neo1-AS2 | TAG AAG AAT GCT ATT GGA GCC ACA G |
| Neo1-AS3 | TTG GGA GTT CGC TGT TGC C |
| Neo2-S1 | GAG GGT GAC TAT CCG ACG GTC |
| Neo2-AS1 | GGG AAA TTG TCA TCA CTG AAA ATG |
| Neo2-AS2 | GGA GTC TGC TGG ACA ACA TGG |
| Neo3-S1 | TAC ATT GGA AGC AGC GAC AGC |
| Neo3-S2 | TGG TCC AGT GTG AAA GAA GCG |
| Neo3-S3 | TAA GAT TGA CTC TGG CCT TCC C |
| Neo3-S4 | ATC AAA TTC ACC GAC ATG AGC G |
| Neo3-S5 | AGA TCT CGT GTC CCT CAG CC |
| Neo3-AS1 | TCG TGT CTG GTA GTA TAA GGC AGC |
| Neo3-AS2 | ATA GCC TTC TTC TGT TTA TCC GC |
| Neo3-AS3 | CCT TCT TGC TGA AGT CGG GG |

1. Hubank M, Schatz DG: **cDNA representational difference analysis: a sensitive and flexible method for identification of differentially expressed genes**. *Methods Enzymol.* 1999, **303**:325-349.
